# Increased CSF Aβ during the very early phase of cerebral Aβ deposition in mouse models

**DOI:** 10.15252/emmm.201505026

**Published:** 2015-05-15

**Authors:** Luis F Maia, Stephan A Kaeser, Julia Reichwald, Marius Lambert, Ulrike Obermüller, Juliane Schelle, Jörg Odenthal, Peter Martus, Matthias Staufenbiel, Mathias Jucker

**Affiliations:** 1 Department of Cellular Neurology, Hertie Institute for Clinical Brain Research, University of TübingenTübingen, Germany; 2 DZNE, German Center for Neurodegenerative DiseasesTübingen, Germany; 3 Department of Neurology, Hospital de Santo António-CHPPorto, Portugal; 4 Novartis Institutes for Biomedical Research, Neuroscience Discovery BaselBasel, Switzerland; 5 Institute of Clinical Epidemiology and applied Biostatistics, University of TübingenTübingen, Germany

**Keywords:** Alzheimer's disease, Aβ, biomarker, CSF, preclinical

## Abstract

Abnormalities in brains of Alzheimer's disease (AD) patients are thought to start long before the first clinical symptoms emerge. The identification of affected individuals at this ‘preclinical AD’ stage relies on biomarkers such as decreased levels of the amyloid-β peptide (Aβ) in the cerebrospinal fluid (CSF) and positive amyloid positron emission tomography scans. However, there is little information on the longitudinal dynamics of CSF biomarkers, especially in the earliest disease stages when therapeutic interventions are likely most effective. To this end, we have studied CSF Aβ changes in three Aβ precursor protein transgenic mouse models, focusing our analysis on the initial Aβ deposition, which differs significantly among the models studied. Remarkably, while we confirmed the CSF Aβ decrease during the extended course of brain Aβ deposition, a 20–30% increase in CSF Aβ40 and Aβ42 was found around the time of the first Aβ plaque appearance in all models. The biphasic nature of this observed biomarker changes stresses the need for longitudinal biomarker studies in the clinical setting and the search for new ‘preclinical AD’ biomarkers at even earlier disease stages, by using both mice and human samples. Ultimately, our findings may open new perspectives in identifying subjects at risk for AD significantly earlier, and in improving the stratification of patients for preventive treatment strategies.

## Introduction

Alzheimer's disease (AD) abnormalities in the brain occur at least 10–20 years before the onset of the first symptoms in both sporadic and familial AD patients (Holtzman *et al*, 2011b; Bateman *et al*, 2012; Buchhave *et al*, 2012). This early stage has been termed ‘preclinical AD’ and is now an important focus of research as it is considered the most promising period for successful disease-modifying therapies (Sperling *et al*, 2013). Thus, a better characterization of this disease stage is crucial for patient stratification (Fagan & Vos, 2013; Jack & Holtzman, 2013).

Disease-specific biomarkers constitute a reasonable approach to defining preclinical AD. Among the most promising biomarkers for characterizing patients at this disease stage are low levels of amyloid-β 42 peptide (the Aβ species that ends with amino acid 42), high levels of Tau protein in cerebrospinal fluid (CSF) (Shaw *et al*, 2009; Bateman *et al*, 2012), atrophy of frontoparietal and temporal regions as detected by magnetic resonance imaging (Mattsson *et al*, 2014), and binding of amyloid-specific ligands using positron emission tomography (PET) (Landau *et al*, 2013; Roe *et al*, 2013). Although the results of these biomarker tests are encouraging in the preclinical stages close to clinical conversion, earlier preclinical stages are not yet satisfactorily captured. Ideally, decade-long, prospective, population-based observational studies are necessary to provide the precise temporal sequence of the different biomarker changes (Jack *et al*, 2013).

Transgenic mice that overexpress human Aβ precursor protein (APP) are useful models for investigating brain Aβ pathology, and recently their translational value for bodily fluid biomarker research has been demonstrated (Jucker, 2010; Tanghe *et al*, 2010; Maia *et al*, 2013). Mouse models allow a direct comparison of brain pathology and biomarkers, which avoids the diagnostic uncertainty present in human preclinical AD cohorts. Moreover, the homogeneity of genetically defined mouse models reduces the inter-individual variability and facilitates the use of mice in a cross-sectional study design.

We previously reported a 50–80% age-related decline in Aβ42, and to a lesser extent in Aβ40 in the CSF of APP transgenic mice (Maia *et al*, 2013). The levels of both peptides were inversely correlated with Aβ deposition in brain, an observation virtually identical to that reported in AD patients (Maia *et al*, 2013). However, our previous study was designed to capture CSF Aβ and total Tau changes with increasing cerebral Aβ deposition and did not allow us to resolve putative CSF changes at the initial phase of Aβ deposition or even before the onset of cerebral β amyloidosis.

We have now studied CSF Aβ changes in three different APP transgenic mouse models, focusing our analysis on the time of the initial Aβ deposition in the brain, which differs significantly among the three mouse models. Remarkably, while we confirmed the CSF Aβ decrease during the later course of brain Aβ deposition, we consistently found a 20–30% increase in CSF Aβ40 and Aβ42 around the time of the appearance of the first Aβ plaques in all three models.

## Results and Discussion

### CSF Aβ40 and Aβ42 exhibit a biphasic profile in APP transgenic mouse models

**Figure 1:**
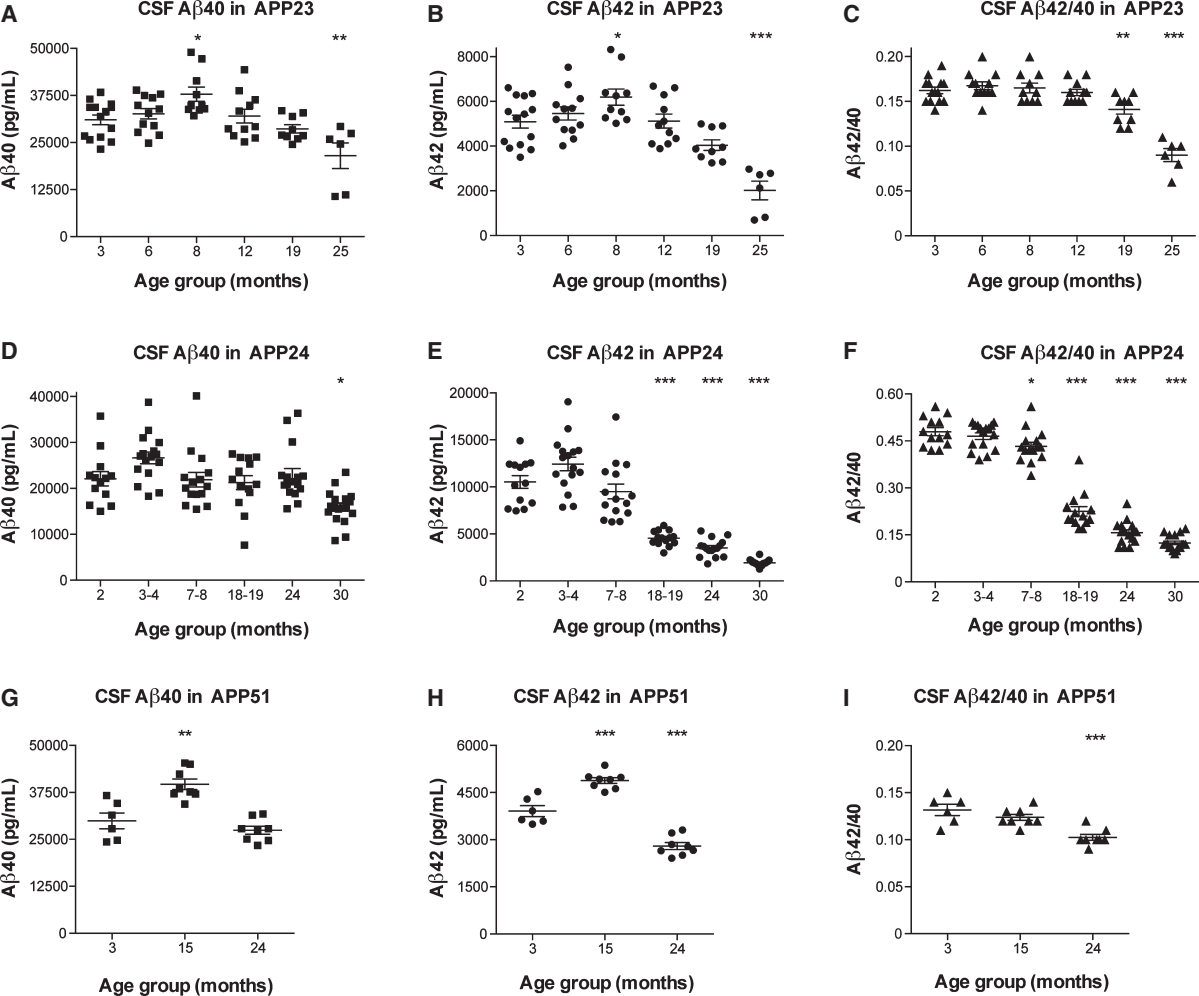
Human Aβ exhibits a biphasic profile in CSF of APP transgenic mice

**Figure 2:**
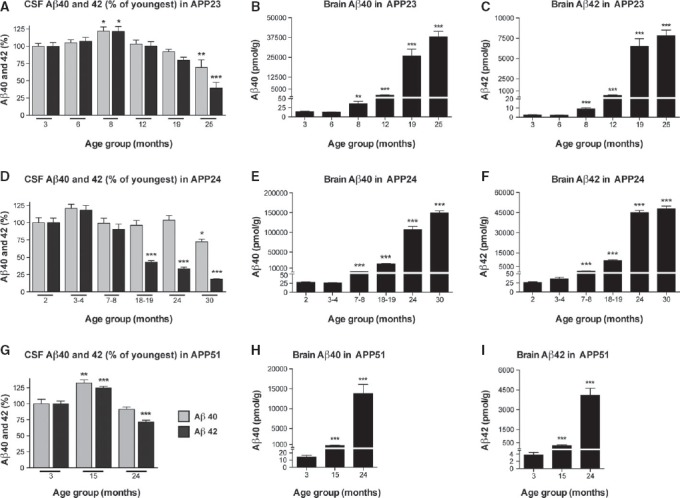
Human Aβ in CSF and brain of APP transgenic mice

APP23 mice expressing human APP with the Swedish mutation were used to test for CSF Aβ40 and Aβ42 changes prior to and during early plaque formation (Sturchler-Pierrat *et al*, 1997). Both Aβ peptides increased in these mice up to 8 months of age, followed by a steady decline that was more pronounced for Aβ42 than for Aβ40 (Fig1A and B). At the peak concentrations (8 months), there was a 22% increase for both CSF Aβ40 (95% CI: 110–134) and Aβ42 (95% CI: 108–136) compared to the 3-month-old group (Fig1A and B). This inverted U-shaped pattern followed a significant quadratic trend for both CSF Aβ40 and Aβ42 (Fig1A and B, see also Fig2A). The CSF Aβ42/40 ratio did not change until 8 months of age but decreased thereafter (Fig1C).

A, B Aβ40 and Aβ42 concentrations in CSF of male APP23 mice (heterozygous; 3 (*n *=* *14), 6 (*n *=* *12), 8 (*n *=* *10), 12 (*n *=* *11), 19 (*n *=* *9), and 25 (*n *=* *6) months of age). CSF Aβ40 (*F*(1, 56) = 22.351, *P < *0.001) as well as CSF Aβ42 (*F*(1, 56) = 38.597, *P < *0.001) followed a significant quadratic trend.

C Aβ42/40 ratio in CSF of APP23 mice showed a delayed decrease with age (*F*(1, 56) = 53.894, *P < *0.001).

D, E Aβ40 and Aβ42 concentrations in CSF of male and female APP24 mice (homozygous; 2 (*n *=* *13), 3–4 (*n *=* *16), 7–8 (*n *=* *15), 18–19 (*n *=* *14), 24 (*n *=* *16), and 30 (*n *=* *18) months of age). CSF Aβ40 followed a significant quadratic trend (*F*(1, 86) = 6.678, *P *=* *0.011) and CSF Aβ42 best fitted a cubic trend (*F*(1, 86) = 30.599, *P < *0.001).

F Aβ42/40 ratio in CSF of APP24 mice showed a delayed decrease with age (*F*(1, 86) = 64.936, *P < *0.001).

G, H Aβ40 and Aβ42 in the CSF of female APP51 mice (heterozygous; 3 (*n *=* *6), 15 (*n *=* *8), and 24 (*n *=* *8) months of age; 22 mice in total). CSF Aβ40 (*F*(1, 19) = 37.349, *P < *0.001) as well as CSF Aβ42 (*F*(1, 19) = 107.670, *P *<* *0.001) followed a significant quadratic trend.

I Aβ42/40 ratio in CSF of APP51 mice showed a delayed decrease with age (*F*(1, 19) = 26.367, *P < *0.001). Data information: *Post hoc* Dunnett's test was employed for group comparisons, which were always conducted between the youngest group and all other groups. (Observed CSF Aβ40 or Aβ42 changes were independent of batch.) All data are represented as group means ± SEM; *P <* *0.05; **P <* *0.01; and ***P <* *0.001.

A APP23 CSF Aβ40 and Aβ42 in the same animals as shown in Fig1. CSF Aβ42 and Aβ40 are expressed as percentages of levels measured in the youngest age group.

B, C Aβ40 and Aβ42 (pmol/g wet brain) in the FA-soluble brain extract from the same APP23 mice showed a robust increase with age; ANOVA revealed a significant cubic trend (*F*(1, 56) = 221.114, *P < *0.001 and *F*(1, 56) = 370.947, *P < *0.001, respectively).

D APP24 CSF Aβ40 and Aβ42 in the same animals shown in Fig1 as percentage of the youngest age group.

E, F Aβ40 and Aβ42 (pmol/g wet brain) in the brain from the same APP24 mice also showed a robust increase with age; ANOVA revealed a significant cubic trend (*F*(1, 86) = 202.173, *P < *0.001 and *F*(1, 86) = 139.941, *P < *0.001, respectively).

G APP51 CSF Aβ40 and Aβ42 in the same animals shown in Fig1 as percentages of levels in the youngest age group.

H, I Aβ40 and Aβ42 (pmol/g wet brain) in the brain from the same APP51 mice showed a robust increase with age; ANOVA revealed a significant quadratic trend (*F*(1, 19) = 12.960, *P *=* *0.002 and *F*(1, 19) = 19.366, *P < *0.001, respectively). Data information: *Post hoc* Dunnett's test group comparisons were always conducted between the youngest group and all other groups. All data are represented as group means ± SEM; *P <* *0.05; **P <* *0.01; and ***P <* *0.001.

To confirm the finding in different models, we used APP24 mice that express human APP with both the Swedish and London mutations (Abramowski *et al*, 2008), as well as APP51 mice, which express human wild-type APP (Bodendorf *et al*, 2002). Homozygous APP24 mice were chosen to obtain roughly similar APP expression, whereas the mutations affect Aβ generation or isoform ratio, and hence the onset of Aβ plaque formation. For both APP24 and APP51 mouse lines, CSF Aβ40 and Aβ42 showed the same inverted U-shaped pattern (Fig1D–I) but, strikingly, peaked at a different age compared to the APP23 model. In the APP24 model, the peak in CSF Aβ occurred at 3–4 months, while in APP51 mice, CSF Aβ was increased at 15 months of age. In APP24 mice, the increase at 3–4 months was 21% for CSF Aβ40 (95% CI: 109–132) and 18% for Aβ42 (95% CI: 105–132) when compared to the 2-month-old age group. In APP51 mice, we observed a 33% rise for CSF Aβ40 (95% CI: 123–142) and 25% for Aβ42 (95% CI: 120–130) at 15 months compared to 3 months of age. Similar to APP23 mice, the CSF Aβ40 and Aβ42 profiles in APP24 and APP51 mice followed a significant cubic and quadratic trend, respectively (see also Fig2D and G).

### Increase in CSF Aβ40 and Aβ42 coincides with the onset of brain Aβ deposition

**Figure 3:**
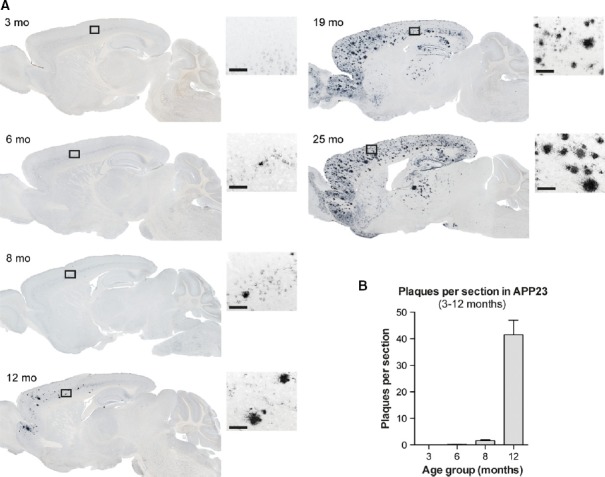
Aβ plaque pathology in the brains of APP23 mice

In largely the same mice as used for CSF measurements, we then analyzed the amount of total brain Aβ by immunoassay and assessed the onset of Aβ deposition by Aβ immunohistochemistry (see Materials and Methods for details). Remarkably, the robust increase in brain Aβ40 and Aβ42 in the APP23 mice started at 8 months, the same age when CSF Aβ40 and Aβ42 peaked (Fig2A–C). Immunohistochemistry revealed that first Aβ plaques appeared at 6 months of age (on average 0.2 plaques per entire sagittal brain section) but it was only at 8 months of age that more than one plaque was present per entire sagittal brain section (on average 1.7 plaques per section) (Fig3).

Aβ immunostaining (CN3 antibody, dark blue) in 25-μm sagittal brain sections shows only sparse Aβ deposits primarily in the frontal cortex of 6- to 8-month-old APP23 mice. At 12 months and thereafter, there is a progressive increase in plaque number and size and a progressive involvement of different brain regions. Insets highlight the plaque characteristics at the different ages. Scale bar, 100 μm.

The mean number of Aβ plaques per section per hemibrain increased with age in 3- to 12-month-old mice. Only four mice were analyzed in the 3-month-old age group, as APP23 mice do not develop plaques at this age (Sturchler-Pierrat *et al*, 1997). The 6-, 8-, and 12-month-old age groups included 12, 10, and 11 mice, respectively (these are the same mice that were used for CSF and brain Aβ measurements). Note that the Aβ plaques became too numerous and often could no longer be individually distinguished in the age groups > 12 months of age. Data are represented as group means ± SEM.

Similar observations were made with the other two mouse lines. For the APP24 mice, a significant increase in brain Aβ40 and Aβ42 by immunoassay was found in the 7–8-month-old group (Fig2E and F); however, it was already at 3–4 months of age that at least 1 plaque was present per sagittal brain section (on average 11.4 plaques per section) and thus coinciding with the increased CSF Aβ level. Because most of these plaques were ‘only’ diffuse in nature, this early deposits may not have been picked up with the immunoassay. In APP51 mice, both immunoassay and immunostaining (on average 3.8 plaques per section) revealed increases at 15 months when CSF Aβ was increased (Fig2G–I). Moreover, in all three models up to the time when CSF Aβ peaked, there was a positive correlation between Aβ levels in brain and Aβ40 and Aβ42 levels in CSF although only significant for the APP51 model (Supplementary Fig S1).

### sAPPβ increases with aging in the APP transgenic mouse lines

**Figure 4:**
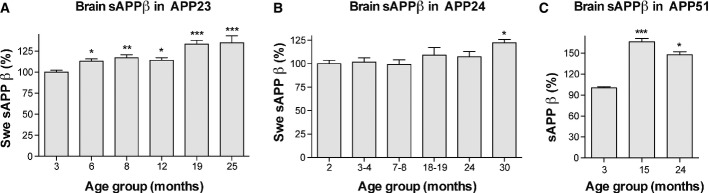
Brain sAPPβ shows an age-related increase in APP23, APP24, and APP51 mice sAPPβ was measured in Triton X-100 brain extracts from largely the same mice as analyzed in Figs1 and 2 and is expressed as percentages of levels measured in the youngest age group.
Swedish sAPPβ showed an age-dependent increase in APP23 mice following a linear trend (*F*(1, 83) = 52.914, *P < *0.001); APP23 from two independent batches were included in this analysis (see Materials and Methods and Supplementary Fig S2 for details).

To determine whether the age-related changes in CSF Aβ concentration may reflect changes in the amyloidogenic APP processing pathway, brain sAPPβ was measured (Bodendorf *et al*, 2002). Overall, we found a significant age-related increase in sAPPβ in all of the models that appeared to be more prominent in APP23 and APP51 mice (Fig4; Supplementary Fig S2). While the changes in sAPPβ did not allow to demonstrate a consistent relation to the onset of plaque formation, it is possible that the initial increase in CSF Aβ40 and Aβ42 is governed by an increase in Aβ generation via the amyloidogenic APP processing pathway. The decline of CSF Aβ that follows the increase (more prominent for Aβ42 than 40) may then be caused by Aβ deposition onto amyloid plaques (sequestering hypothesis). As plaques and their Aβ binding sites increase, Aβ sequestration also goes up and eventually outbalances the increase in Aβ with aging in all the models. This then leads to the decline of soluble Aβ that reaches the CSF.

Swedish sAPPβ showed an age-dependent increase in APP24 mice following a linear trend (*F*(1, 84) = 11.264, *P *=* *0.001).

Human wild-type sAPPβ showed an age-dependent increase in APP51 following a quadratic trend (*F*(1, 18) = 68.980, *P < *0.001).

Data information: *Post hoc* Dunnett's test group comparisons were always conducted between the youngest group and all other groups. All data are represented as group means ± SEM; *P <* *0.05; **P <* *0.01; and ***P <* *0.001. For absolute values, see Supplementary Fig S2.

### Broadening the preclinical AD concept

The concept of ‘preclinical AD’ is challenging because it relies largely on biochemical and imaging biomarkers, without neuropathological confirmation (Blennow *et al*, 2010; Jack & Holtzman, 2013). The most prominent early biomarkers are low levels of Aβ42 in CSF and brain retention of amyloid-binding ligands using PET both reflecting brain deposition of Aβ. Studies of long-term longitudinal changes in these biomarkers are still lacking (Roe *et al*, 2013; Toledo *et al*, 2013; Fagan *et al*, 2014). Even more important, both low CSF Aβ and brain retention of amyloid-binding ligands are apparent only after substantial Aβ deposition in the brain (Ikonomovic *et al*, 2012). Thus, how biomarkers change before a considerable amount of Aβ has already been deposited remains unknown (Tapiola *et al*, 2009; Jack *et al*, 2013). It is therefore crucial to elucidate the trajectories of these earliest biomarker changes in order to identify subjects at risk, monitor disease progression, and, ultimately, characterize the effects of early therapeutic interventions (Jack *et al*, 2013; Fagan *et al*, 2014).

In this study, we sought to reveal such initial biomarker changes using a set of cerebral β-amyloidosis mouse models over-expressing mutated and wild-type human APP (Sturchler-Pierrat *et al*, 1997; Bodendorf *et al*, 2002; Abramowski *et al*, 2008). We took advantage of the different onset of Aβ deposition among the three models used to show that CSF Aβ40 and Aβ42 levels exhibited a significant inverted U-shaped profile that peaked when the first Aβ plaques appeared. In fact, although the age when the increase was observed varied from 3 to 4 months in APP24 mice to 8 months in APP23 mice and 15 months in APP51 mice, the increase in CSF Aβ consistently coincided with the emergence of deposits in the different mouse lines.

The increase in CSF Aβ40 and Aβ42 ranged from 20 to 30% when compared to the levels determined at the youngest age in each of the models. Remarkably, in the most recent cross-sectional biomarker analysis of the dominantly inherited AD network (DIAN) study, AD mutation carriers revealed a similar, approximately 20% increase in CSF Aβ40 15–20 years before the predicted age of clinical onset (Fagan *et al*, 2014). This increase occurred 5–10 years before the classic biomarker changes associated with the Aβ pathology became apparent (CSF Aβ42 decrease and positivity in amyloid PET scans). Given the present findings in the mouse models, it is appealing to suggest that the increase in CSF Aβ40 may indeed reflect the onset of AD plaque deposition in these patients. Unlike our findings, in the AD mutation carriers, CSF Aβ42 did not show a corresponding increase. However, DIAN includes subjects with different mutations (APP, presenilin 1, and presenilin 2) characterized by a heterogeneous over-production of Aβ42, which may have masked any transient increase (Scheuner *et al*, 1996; Bateman *et al*, 2012; Potter *et al*, 2013). Alternatively, the increase in CSF Aβ42 may occur at even earlier ages, a possibility that has not yet been addressed in the DIAN study.

After the peak, we observed a consistent decrease in CSF Aβ42 in all three models that correlated inversely with the increase in brain Aβ deposition. This was particularly notable in APP24 mice followed by APP23 mice, as these models deposited considerably more Aβ when compared to APP51 mice. This observation is consistent with previously published work on mouse models of β-amyloidosis (Kawarabayashi *et al*, 2001; Hong *et al*, 2011; Maia *et al*, 2013). It is also in line with what is predicted to occur in human sporadic and familial AD patients, supporting the concept that once brain Aβ deposition spreads, soluble Aβ42 is sequestered in the plaques and, consequently, reduced in the CSF (Blennow *et al*, 2010; Holtzman *et al*, 2011a; Bateman *et al*, 2012). Our data suggest that the CSF Aβ peak may antedate the CSF Aβ drop by a relatively long period of time. A similar peak may be missed in preclinical AD patients, as long intervals before the available biomarker changes (decrease in CSF Aβ42, positive amyloid tracer PET) are not analyzed. Importantly, as the observed CSF Aβ profile is biphasic, identical CSF Aβ concentrations may correspond to different pathological stages. This implies that preclinical patient stratification solely based on this biomarker, could be misleading. Presently, familial AD patients are stratified based on predicted age of onset of mutation carriers and sporadic preclinical AD patients are identified based on amyloid positive markers precluding preventive treatment trials. Longitudinal analysis of CSF Aβ and identification of other biomarkers defining this disease stage would certainly increase the possibility of an earlier and timely preventative treatment in better stratified patients.

To address the potential mechanism underlying the initial CSF Aβ increase, we measured sAPPβ in the brain (Bodendorf *et al*, 2002). In all models, sAPPβ increased with age, reflecting a possible increase in APP processing via the amyloidogenic pathway. Indeed, an age-related increase in BACE activity in brain has been shown to occur across different species (Fukumoto *et al*, 2004; Pera *et al*, 2013). Our finding suggests that an increase in Aβ production may contribute to the initial increase in CSF Aβ until plaque deposition occurs. However, additional explanations for the present findings, such as insufficient Aβ clearance with aging as well as an age-related increase in the half-life of sAPP, cannot be excluded (Dewachter *et al*, 2000; Mawuenyega *et al*, 2010).

Overall, we have shown that CSF Aβ40 and Aβ42 exhibit a biphasic profile in murine models of cerebral β-amyloidosis. Most importantly, in three transgenic mouse lines, we linked the transient increase in CSF Aβ peptides to the age at which Aβ plaques emerge. Mechanistically, the observed CSF Aβ changes seem to be governed distinctively: during the first phase by the increase in Aβ and during the second phase by Aβ sequestration in the brain deposits, outbalancing the increased amyloidogenic APP processing especially for Aβ42. The evidence obtained in the three APP transgenic mouse models is compelling and holds potential to be translated to both late onset AD and dominantly inherited AD. Indeed, initial hints from earlier publication (Shoji *et al*, 2001) and more recent in dominantly inherited AD (Fagan *et al*, 2014) suggest that CSF Aβ levels may also increase in early preclinical sporadic AD patients prior to the well-known decline at later stages. Together with the present findings in the mice, this will hopefully stimulate the search for similar changes in the ongoing longitudinal studies and to address their potential as biomarkers. If confirmed, a CSF Aβ peak would probably take place 20–25 years prior to clinical symptoms and would be the ideal timing to start primary prevention for AD.

In short, our observations will hopefully pave the way to an even earlier detection of presymptomatic individuals and a better stratification of patients for clinical trials of preventive treatments for AD.

## Materials and Methods

### APP23 mice

Male 3- to 25-month-old heterozygous APP23 mice (Sturchler-Pierrat *et al*, 1997) were all bred at the Hertie Institute for Clinical Brain Research (Tübingen, Germany). APP23 mice express the K670M/N671L-mutated human APP (Swedish double mutation) under control of the neuron-specific Thy1 promoter element at about 7-fold over endogenous (murine) APP. The mice were generated on a B6D2 background, but have since been bred with C57BL/6J mice for over 20 generations. APP23 mice have been reported to develop plaques beginning at 6–8 months of age, and plaque development is faster in females than in males (Sturchler-Pierrat *et al*, 1997; Eisele *et al*, 2010). For the present study, only male animals were used to minimize variability and reduce sample size. All mice were kept under specific pathogen-free conditions. The experimental procedures were conducted in accordance with the veterinary office regulations of Baden-Württemberg (Germany) and were approved by the local Animal Care and Use Committees.

### APP24 mice

Male and female 2- to 30-month-old homozygous APP24 mice (Abramowski *et al*, 2008) were bred at both the Novartis Mouse facility (Basel, Switzerland) and the Hertie Institute for Clinical Brain Research (Tübingen, Germany). The first colony was used for Aβ assessment in CSF and assessment of Aβ and sAPPβ in brain. The second colony was used for histological studies, as there were no fixed brains available from the initial (Basel) cohort. APP24 mice are on a C57BL/6J background and express K670M/N671L- and V717I (London)-mutated human APP, the latter of which increases the Aβ42/40 ratio. Expression is under control of the neuron-specific Thy1 promoter element, and in homozygous mice, it is about 7-fold over endogenous (murine) APP. Homozygous APP24 mice develop the first plaques between 3 and 4 months of age without a prominent gender difference. The experimental procedures were conducted in accordance with the veterinary office regulations of Basel (Switzerland) and Baden-Württemberg (Germany) and were approved by the local Animal Care and Use Committees.

### APP51 mice

Female 3- to 26-month-old heterozygous APP51 mice (Bodendorf *et al*, 2002) were bred at both the Novartis Mouse facility (Basel, Switzerland) and the Hertie Institute for Clinical Brain Research (Tübingen, Germany). The first colony was used for Aβ assessment in CSF and for the assessment of Aβ and sAPPβ in brain. The second colony was used for histological studies, as there were no fixed brains available from the initial (Basel) cohort. APP51 mice express the human wild-type APP under control of the neuron-specific Thy1 promoter element at about 7-fold over endogenous (murine) APP and were bred on a C57BL/6J background. APP51 mice develop the first plaques between 13 and 15 months of age. The experimental procedures were conducted in accordance with the veterinary office regulations of Basel (Switzerland) and Baden-Württemberg (Germany) and were approved by the local Animal Care and Use Committees.

### CSF collection and tissue harvesting

CSF collection was undertaken as described previously adopting a standardized protocol for CSF collection matching human QC protocols (Maia *et al*, 2013). Briefly, CSF was collected at a fixed time-point to minimize circadian CSF Aβ variations (Kang *et al*, 2009). After anesthetizing the mice, CSF was immediately collected from the cisterna magna. CSF samples were then centrifuged at 13,000 *g* for 30 s, assessed macroscopically for blood contamination, aliquoted (5 μl), and stored at −80°C until use. Blood-contaminated samples were not analyzed. Thereafter, mice were perfused with ice-cold sterile PBS. The brain was removed, and one hemibrain (left) was snap-frozen in dry ice and stored at −80°C until use. The other hemibrain (right) was fixed in 4% paraformaldehyde with 0.1 M PBS, pH 7.6, for 48 h at 4°C, immersed in 30% sucrose for an additional 24 h at 4°C, snap-frozen in 2-methylbutane, and stored at −80°C.

### Biochemical analysis of brain tissue

Hemibrains from APP23 mice were homogenized at 10% (w/v) in homogenization buffer (50 mM Tris pH 8.0, 150 mM NaCl, 5 mM EDTA, and Complete protease inhibitor cocktail from Roche Molecular Biochemicals) on ice using a Dounce (IKA, Staufen, Germany) or Precellys (Bertin, Montigny-le-Bretonneux, France) homogenizer. The homogenized brain tissue was aliquoted and stored at −80°C until use. For Aβ measurements, the homogenates were extracted as follows: Aliquots were thawed on ice, mixed 1:3.2 with cold formic acid (FA) (min. 96% purity, Sigma, St. Louis, MO, USA), sonicated for 35 s at 4°C, and spun at 25,000 *g* at 4°C for 1 h. The supernatant was collected as the ‘FA-soluble fraction’ and equilibrated (1:20) in neutralization buffer (1 M Tris base, 0.5 M Na_2_HPO_4_, 0.05% NaN_3_). The brain tissue from the APP24 and APP51 mice was similarly prepared with the following deviations: First, forebrains (hemibrains without the cerebellum) were used, and second, homogenization was done at 10% (w/v) in TBS (30 mM Tris–HCl pH 7.6, 137 mM NaCl, Complete protease inhibitor cocktail, Roche) by vigorous shaking with metal beads in a Retsch mill, followed by brief sonication.

For sAPPβ measurements, we used Triton X-100 (Sigma, St. Louis, MO, USA) extracts as previously described (Abramowski *et al*, 2008). In brief, the homogenates were thawed on ice, mixed 1:1 with 2% Triton X-100–TBS solution with regular vortexing for 15 min, and spun at 20,800 *g* at 4°C for 15 min, and finally the supernatants were collected as the ‘Tx-soluble brain extracts’ for analysis.

### Electrochemiluminescence-linked immunoassay for Aβ in CSF and brain extracts

Aβ concentrations in CSF and brain extracts from APP transgenic mice were determined with an electrochemiluminescence-linked immunoassay using the MSD® 96-well MULTI-SPOT® Human (6E10) Aβ Triplex Assay (Meso Scale Discovery, Gaithersburg, MD, USA). CSF was analyzed according to the manufacturer's instructions, as described previously (Maia *et al*, 2013). Brain Aβ detection was done in Aβ triplex plates. FA-soluble brain samples were diluted 1:10 to 1:100 in dilution buffer and measured. Measurements were performed by a blinded researcher (ML or JR). Data analysis used MSD® DISCOVERY WORKBENCH® software 2.0. Every sample was tested in duplicate, and those with a coefficient of variance (CV) over 20% were excluded from the analysis or repeated if additional material was available. Internal reference samples were used as a control in every plate, and the results were adjusted for inter-plate variability. Assay performance was within the standards of biomarker measurements, and inter-plate CVs for the different analytes were < 15% (Aβ40 inter-plate CV = 12%; Aβ42 inter-plate CV = 15%).

### Electrochemiluminescence-linked immunoassay for secreted APP (sAPP) beta in Triton X-100 brain extracts

Wild-type sAPPβ in APP51 brain samples and Swedish sAPPβ in APP23 and APP24 brain samples were determined with an electrochemiluminescence-linked immunoassay using the MSD® 96-well MULTI-SPOT® Human sAPPβ or Swedish sAPPβ assay (Meso Scale Discovery, Gaithersburg, MD, USA). The brains analyzed are from the same animals that had the CSF Aβ measured. In the APP23 model, we used an additional batch of mice to confirm the findings from the original set of APP23 mice. ‘Tx-soluble brain extracts’ were diluted up to 1:100,000 in blocking buffer containing 1% Triton X-100 (in order to stay within the linear range of the assay). Data analysis used MSD® DISCOVERY WORKBENCH® software 2.0. Every sample was tested in duplicate, and those with a coefficient of variance (CV) over 20% were excluded from the analysis or repeated. Internal reference samples were used as a control in every plate, and the results were adjusted for inter-plate variability.

### Histology and immunohistochemistry

After freezing, fixed brains were cut into serial, 25-μm-thick sagittal sections using a freezing–sliding microtome. The sections were collected in 0.1 M Tris-buffered saline (pH 7.4) and stained immunohistochemically according to previously published protocols using anti-Aβ polyclonal antibody CN3 (Maia *et al*, 2013).

### Quantification of total Aβ plaque load

Aβ plaque load was quantified on an Aβ immunostained set of every 12^th^ systematically sampled, serial, sagittal section throughout the entire brain, except for 5 APP51 mice from the 15-month age group, which were sectioned coronally due to processing error. Aβ immunostained plaques were counted manually using a 10× objective (0.30 numerical aperture) and a Zeiss Axioskop 2 microscope (Zeiss, Oberkochen, Germany).

**The paper explained**

**Problem**

It is now widely recognized that abnormalities in the brain of AD patients start long before the first clinical symptoms emerge. It is also consensual in the field that future drug trials need to be performed at an earlier stage of the disease and that biomarkers are essential to guide such trials. However, little is known about early biomarkers and their dynamics in the very initial disease stages limiting preclinical treatment approaches.

**Results**

We analyze three APP transgenic mouse models that differ significantly in the age of onset of Aβ plaque pathology. Remarkably, a temporary and consistent 20–30% increase in CSF Aβ was found just at the time of the appearance of the first individual Aβ plaques in all three models. Mechanistically, the CSF Aβ biphasic changes seem to be governed distinctively: during the first phase by an increase in Aβ generation and during the second phase by Aβ sequestration in the brain deposits, outbalancing the increased amyloidogenic APP processing.

**Impact**

These unexpected results hold great potential to be directly translated and of immediate importance to humans. The biphasic nature of the observed biomarker changes may indicate a different concept of CSF Aβ dynamics and further stresses the need for longitudinal biomarker studies in the clinical setting. Moreover, as identical CSF Aβ concentrations may correspond to different pathological stages, the search for new ‘preclinical AD’ biomarkers at even earlier disease stages becomes highly pertinent by using both mice and human samples. Ultimately, our findings may open new perspectives in identifying subjects at risk for AD significantly earlier, and in improving the stratification of patients for preventive treatment strategies.

### Statistical analysis

The distribution of quantitative data was assessed analyzing Q–Q plots and confirmed by the Kolmogorov–Smirnov test. Non-normally distributed variables were logarithmic-transformed. To examine whether CSF and brain Aβ levels change with aging in APP transgenic mice, a trend test derived from an ANOVA was calculated. The primary prespecified analysis was whether a linear trend in CSF and brain Aβ levels depending on age was present. Additionally, to improve fit, a quadratic term was investigated exploratory. Only in case of significant linear trend, subsequent special pairwise comparisons were done. This is in accordance with the principal of hierarchically ordered hypotheses. Only differences between the youngest APP transgenic mouse group and all other age groups were analyzed using Dunnett's *post hoc* test for multiple comparisons of the youngest age group to all the others. Correlation analysis was done using Spearman's or Pearson's correlation coefficient, depending on the bivariate visual distribution of the data. Values are mean ± SEM, unless specified. Statistical tests were justified for each figure, as appropriate. In all cases, statistical significance was set at *P *<* *0.05. SPSS version 22 was used for statistical analysis, and Graphpad Prism version 5 was used to generate the graphics.

## Supporting Information

Supplementary Figures

Review Process File
