# Supplementary material for: Increased CSF Aβ during the very early phase of cerebral Aβ deposition in mouse models
Source: EMBO Mol Med. 2015 May 15;7(7):895–903. doi: 10.15252/emmm.201505026 (PMC4520655; doi:10.15252/emmm.201505026)
Supplement: Supplementary file 1 [file emmm0007-0895-sd1.pdf]

Supplementary Information

Increased CSF Aβ during the very early phase of cerebral Aβ deposition in mouse models

Luis F. Maia[1,2,3], Stephan A. Kaeser[1,2], Julia Reichwald[4], Marius Lambert[1,2], Ulrike Obermüller[1,2], Juliane Schelle[1,2], Jörg Odenthal[1,2], Peter Martus[5], Matthias Staufenbiel[1,2,4], Mathias Jucker[1,2]

[1]Department of Cellular Neurology, Hertie Institute for Clinical Brain Research, University of Tübingen, D-72076 Tübingen, Germany; [2]DZNE, German Center for Neurodegenerative Diseases, D-72076 Tübingen, Germany; [3]Department of Neurology, Hospital de Santo António-CHP, 4099-001 Porto, Portugal; [4]Novartis Institutes for Biomedical Research, Neuroscience Discovery Basel, CH-4056 Basel, Switzerland. [5]Institute of Clinical Epidemiology and applied Biostatistics, University of Tübingen, D-72076 Tübingen, Germany.

Content:

Supplementary Figure S1
Supplementary Figure S2

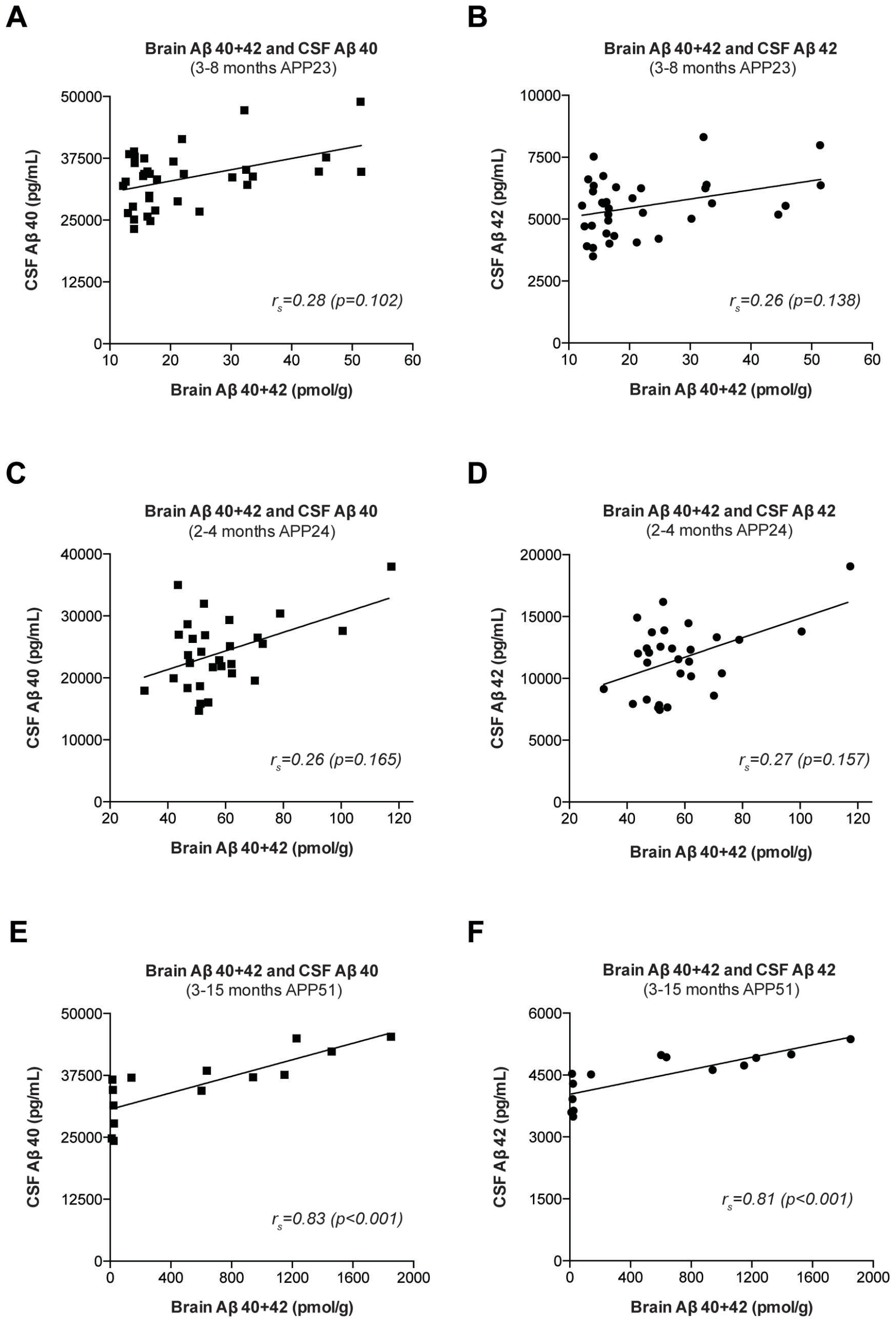

Supplementary Figure S1. *Correlation analysis of brain Aβ40+42 concentrations versus CSF Aβ40 and Aβ42 in early stage APP23, APP24 and APP51 mice.* We observed a positive correlation between CSF Aβ40 and Aβ42, and brain Aβ40+42 (measured by ECL based assay) in the early stages of Aβ deposition in the 3 models. (**A and B**) 3 to 8 month-old APP23 mice, (**C and D**) 2- to 4-month-old APP24 mice and (**E and F**) 3- to 15-month-old APP51 mice. (r_s = Spearman correlation coefficient). *p* values are shown for each of the observed correlations.

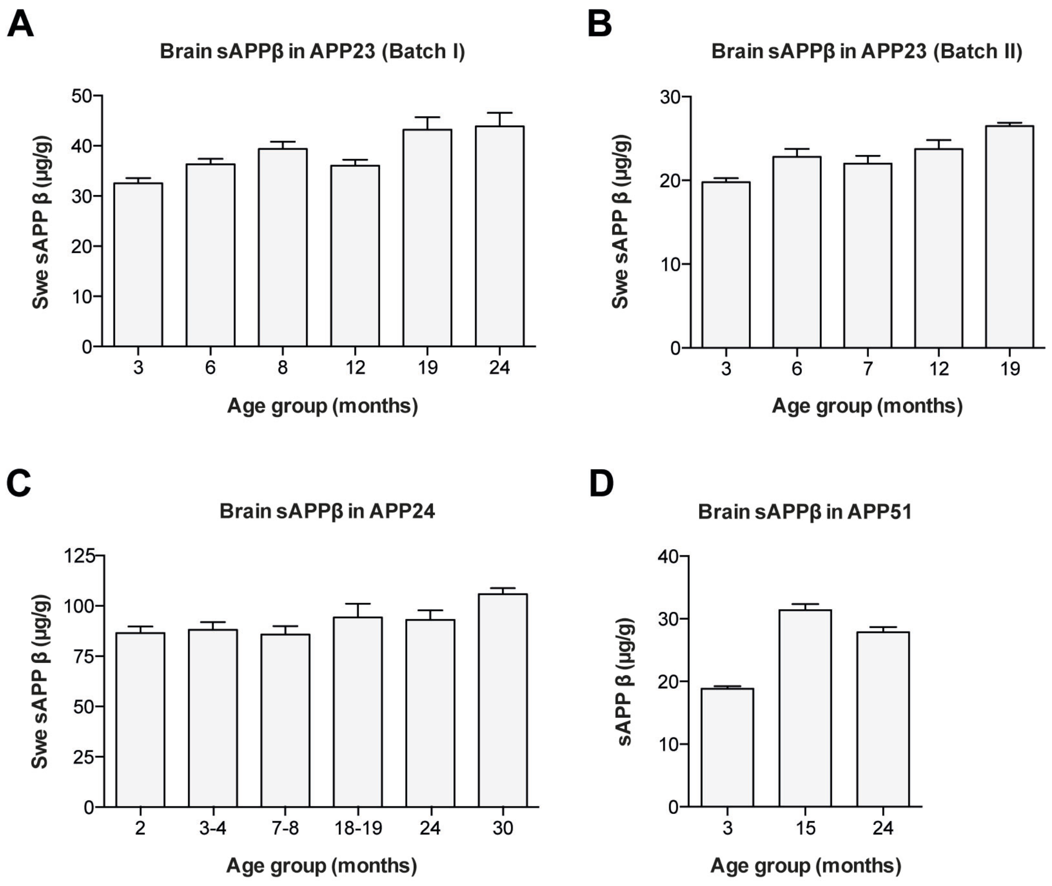

Supplementary Figure S2 – *Brain sAPPβ shows an age-related increase in APP23, APP24 and APP51 mice.* Shown are the absolute values of brain sAPPβ underling Figure 4. (**A and B**) In APP23 our initial analysis (shown in **A**) revealed an age-dependent increase of Swedish sAPPβ following a linear trend ($F(1,54)=29.865$, $p<0.001$). However, sAPPβ levels at 12 month seemed slightly off the linear trend. To confirm such finding we have analyzed a second batch of APP23 mice (shown in **B**). While we confirmed sAPPβ increase with age following a linear trend ($F(1,24)=30.195$, $p<0.001$) the decrease at 12 months was no longer evident. Due to a technical incident the measured values in the two batches could not be pooled and for that reason results were plotted as % of the youngest age group in Fig. 4. **(C)** Swedish sAPPβ showed an age-dependent increase in APP24 mice following a linear trend ($F(1,84)=11.130$, $p=0.001$)**;** To be consistent the data for APP24 in Fig. 4 are also shown as % of the youngest age group. **(D)** Human wild-type sAPPβ showed an age-dependent increase in APP51 following a quadratic trend ($F(1,18)=67.021$, $p<0.001$). Again, for consistency the data for APP51 in Fig. 4 are shown as % of the youngest age group.
