# Supplementary material for: Increased CSF Aβ during the very early phase of cerebral Aβ deposition in mouse models
Source: EMBO Mol Med. 2015 May 15;7(7):895–903. doi: 10.15252/emmm.201505026 (PMC4520655; doi:10.15252/emmm.201505026)
Supplement: Supplementary file 2 [file emmm0007-0895-sd2.pdf]

EMBO Molecular Medicine

Increased CSF Aβ during the very early phase of cerebral Aβ deposition in mouse models

Luis F. Maia, Stephan A. Kaeser, Julia Reichwald, Marius Lambert, Ulrike Obermüller, Juliane Schelle, Jörg Odenthal, Peter Martus, Matthias Staufenbiel, Mathias Jucker

Corresponding author: Mathias Jucker, University of Tübingen

Review timeline:

Submission date:	16 January 2015
Editorial Decision:	09 February 2015
Revision received:	23 March 2015
Editorial Decision:	13 April 2015
Revision received:	20 April 2015
Accepted:	21 April 2015

Transaction Report:

(Note: With the exception of the correction of typographical or spelling errors that could be a source of ambiguity, letters and reports are not edited. The original formatting of letters and referee reports may not be reflected in this compilation.)

Editor: Céline Carret

1st Editorial Decision 09 February 2015

Thank you for the submission of your manuscript to EMBO Molecular Medicine. We have now heard back from the two referees whom we asked to evaluate your manuscript. Although the referees find the study to be of potential interest, they also raise a number of concerns that must be addressed in the next final version of your article.

As you will see, both reviewers appreciate the work and use of three different mouse models. However, while referee 2 is fully supportive, referee 1 is more reserved and besides technical issues, particularly emphasises that more work on BACE1 is needed to strengthen the data and support the conclusions. In addition, more discussion on the translational potentials of the findings seems to be needed.

Given the balance of these evaluations, we feel that we can consider a revision of your manuscript if you can address the issues that have been raised. Please note that it is EMBO Molecular Medicine policy to allow only a single round of revision and that, as acceptance or rejection of the manuscript will depend on another round of review, your responses should be as complete as possible.

EMBO Molecular Medicine has a "scooping protection" policy, whereby similar findings that are published by others during review or revision are not a criterion for rejection. Should you decide to submit a revised version, I do ask that you get in touch after three months if you have not completed it, to update us on the status.

Please also contact us as soon as possible if similar work is published elsewhere. If other work is published we may not be able to extend the revision period beyond three months.

Please see important information for submission of your revised article below.

I look forward to seeing a revised form of your manuscript as soon as possible.

***** Reviewer's comments *****

Referee #1 (Remarks):

The manuscript by Maia et al provides data that suggest that in APP transgenic mouse models roughly co-incident with the initiation of plaque deposition there is a transient but fairly small rise (~20%) in Abeta40 and Abeta42 followed by a decline in both species but with more marked decline in Abeta42. These are technically challenging studies and the one of the strengths of the manuscript is the use of multiple APP models. Indeed, the authors should be lauded for undertaking and executing this set of challenging studies.
The decline in CSF Abeta has been documented by this group and others before, and the selective/preferential decline in 42 is certainly reflective of human AD. The authors try to link this mechanistically to increase in Beta secretase activity inferred by a measurement of sAPPbeta in the brain during aging, but that data is not convincing, as the timing really only correlates well in one model. Though it is well established that Beta-secretase does accumulate in APP mice and human brain, this is likely due to accumulation of BACE1 within dystrophic neurites and the evidence for this resulting in increased BACE1 cleavage is not strong. Further though this is an intriguing finding without a strong mechanistic link establishing whether this transient increase reflects some sore of impaired clearance or increase in production, then the finding is essentially descriptive.
Extended the translational implications of this effect if it would be reflected in humans is challenging. It's a small increase that would be predicted to be observed some 15-25 years before onset of AD in humans, and we are not likely going to do serial taps in the general population to track this change. Further the likelihood of a 20% increase being observable in humans as opposed to a homogenous inbred population of mice is questionable. Certainly one should be able to track declining Abeta42 in CSF as some indicator of ongoing deposition. But to detect the bump up at onset in humans seems a bit of reach. There are some other significant technical concerns that also need to be clarified.

1. How were the ELISAs normalized were they all run at once or were the samples run in batches? With this kind of small difference batch effects could account for the entire effect.
2. There is no normalization to a protein that should not be altered to effects on proteostasis. Maybe this reflects altered CSF dynamics rather than specific alterations in Abeta production or clearance. The same holds for sAAPbeta, what happned to sAPPalpha for example. What about holo APP, etc..
3. I am a little uncomfortable with the way the statistics is described, though the ANOVA with Dunnet's in Figure 2 is appropriate and perhaps the most important. However, in the methods it sounds as if a whole bunch of various methods were applied. I think it important to be clear on the a priori principal test and outcome versus what was post-hoc analysis.
4. What happened in blood of mice? As these mice use the Thy-1 promoter blood might track better with CNS than other lines using promoters with peripheral expression. Potential translational impact would increase if the changes could be tracked in blood.

Referee #2 (Comments on Novelty/Model System):

This work spears to be of high technical quality and uses multiple mouse models of beta amyloidosis.

Referee #2 (Remarks):

In this manuscript, Maia and colleagues assess CSF levels of Abeta 40 and 42 over the lifespan of 3 different types of APP Tg mice that develop Abeta plaques at differing ages. As this group has previously shown, once there is significant Abeta deposition, both Abeta42 in particular but also Abeta40 drops in CSF, very similar to what is seen in humans that develop amyloid deposition.

What is new in this work is the finding that Abeta42 and Abeta40 increase just around the onset of Abeta plaque deposition in all 3 models. Subsequently, the levels of both species decreases back to what is found in earlier ages, followed by the expected decline with more increasing plaque deposition. The finding that sAPPbeta increases at about the time there is an increase in Abeta in CSF suggests that increased Abeta production might be responsible for this increase. These findings are important in that they suggest the possibility in both late onset AD as well as in dominantly inherited AD, that there may be a transient increase in CSF Abeta species that occurs. This has really not been examined in humans yet but this study strongly suggests that this should now occur in current and future longitudinal studies. The findings would suggest that if there is a similar increase in human AD, it would occur just with the onset of Abeta deposition. This is probably about 20-25 years prior to any cognitive decline. In some of the ongoing studies that this would be worth looking for (such as DIAN or API), this would mean individuals would have to start being assessed as the early to late adolescent period to pick up levels prior to any transient "increase". In normal populations, individuals would have to start being assessed in their 30's since in some populations such as E4/E4 individuals, CSF Abeta appears to start dropping by age 50. In any case, this manuscript highlights this important new finding and argues human studies should look for the same change since primary prevention to block Abeta deposition would need to start prior to this time.

1st Revision - authors' response 23 March 2015

Referee: 1

Main Comment 1: *The decline in CSF Abeta has been documented by this group and others before, and the selective/preferential decline in 42 is certainly reflective of human AD. The authors try to link this mechanistically to increase in Beta secretase activity inferred by a measurement of sAPPbeta in the brain during aging, but that data is not convincing, as the timing really only correlates well in one model. Though it is well established that Beta-secretase does accumulate in APP mice and human brain, this is likely due to accumulation of BACE1 within dystrophic neurites and the evidence for this resulting in increased BACE1 cleavage is not strong. Further though this is an intriguing finding without a strong mechanistic link establishing whether this transient increase reflects some sore of impaired clearance or increase in production, then the finding is essentially descriptive.*

Response 1: It seems we have not expressed our point sufficiently clear and, therefore, changed the text as indicated below. We have found that CSF Abeta 40 and 42 exhibit a biphasic profile with aging in the APP-tg mice. The solidity of our observation relies on its consistency across the three models analyzed; a quadratic trend was significant in every mouse model studied. As stated in the discussion (page 9), the additive effect of different mechanisms likely underlies the biphasic nature of the curve:

(1) The initial increase in CSF Abeta seems to be governed by an increase in Abeta generation via the amyloidogenic APP processing pathway. Such a view is supported by the age-related increase in brain sAPPbeta (Figure 4; Suppl Figure 2) and further by the positive correlations observed between CSF Abeta and sAPPbeta in pre-plaque stage brain (see Figure 1 below; not included in the manuscript since we feel it does not substantially add to the conclusion {unpublished data removed upon author request}).

(2) The decline that follows the increase (more prominent for CSF Abeta 42 than 40) seems to be caused by Abeta deposition onto amyloid plaques (sequestering hypothesis). As plaques and their Abeta acceptor sites increase, Abeta sequestration also goes up and eventually outbalances the increase in Abeta generation present with ageing in all the models. This leads to the decline of soluble Abeta that reaches the CSF.

We agree with the reviewer that other mechanisms may also contribute to the biphasic change but this remains speculative as we did not find such evidence. In particular Abeta clearance to the blood

may change. However, our data from the APP23 model (please see answer to Technical question 4 below) suggest that brain to blood clearance may be insufficient to compensate the increase in Abeta generation during the initial stages (at least for the APP23 model).

As indicated above we have now rephrased these points on page 7 of the revised manuscript and believe the description of our results and the interpretation are more clear now.

Main Comment 2: *Extended the translational implications of this effect if it would be reflected in humans is challenging. It's a small increase that would be predicted to be observed some 15-25 years before onset of AD in humans, and we are not likely going to do serial taps in the general population to track this change. Further the likelihood of a 20% increase being observable in humans as opposed to a homogenous inbred population of mice is questionable. Certainly one should be able to track declining Abeta42 in CSF as some indicator of ongoing deposition. But to detect the bump up at onset in humans seems a bit of reach.*

Response 2: We agree with this referee regarding the current status of the field but believe it should also be considered that progress frequently requires multiple steps and assay quality often is remarkably improved once it becomes urgent as with the availability of a therapy (see also comments and suggestions of referee 2). In this context we would like to point out that serial (longitudinal) CSF analysis is already performed prior to disease onset in several studies such as DIAN or API. Furthermore, the most recent published cross-sectional data from the DIAN study (Fagan et al STM 2014) suggest the possibility of a detectable and significant increase in CSF Abeta 40 in familial AD mutation carriers 20 years before the predicted age of clinical onset. Together with our findings this will hopefully stimulate to search for similar changes in longitudinal studies and to address their potential as biomarkers.

We do agree that a 20-30% increase or decrease will not be easy to track/identify. As an alternative, additional biomarkers may be used to differentially identify patients at such stages. Moreover, one of the important implications from our results is that similar Abeta levels accounted for different stages of Abeta deposition, which further calls for additional biomarkers. On the long run, we expect that an improvement of assay platforms and more strict cut-off values will be attained. Such technical improvements together with longitudinal biomarker measurements (today in the CSF and in the future hopefully in blood) may help to better stratify patients and allow timely preventive treatments.

We have now detailed the translational implications and limitations in the discussion of our manuscript on page 9-10.

Technical concern 1: *How were the ELISAs normalized were they all run at once or were the samples run in batches? With this kind of small difference batch effects could account for the entire effect.*

Response to technical concern 1: Given the number of samples involved and the fact that all measurements were done in duplicate, we had to run the analysis in different batches. So, in order to minimize any bias associated with (inter-)plate variability, in each plate the samples from animals of different ages were strictly balanced. Furthermore, we adjusted the measurements for inter-plate variability using an internal control (stated in methods). We further confirmed that observed CSF Abeta 40 or 42 variation with age was not driven by any specific batch. All CSF sample analyses were conducted as blind experiments, in duplicate and only measurements with CVs < 20% were considered. Assay performance was within the standards for biomarker measurements (Mattson et al., Alzheimers Dement 2011) and inter-plate CVs for the different analytes were <15% (Abeta 40 inter-plate CV=12%; Abeta 42 inter-plate CV=15%). We have now detailed these procedures in the Method part (*Electrochemiluminescence-linked immunoassay for Aβ in CSF and brain extracts*) on page 14 and Figure 1 legend.

Technical concern 2: There is no normalization to a protein that should not be altered to effects on proteostasis. Maybe this reflects altered CSF dynamics rather than specific alterations in Abeta production or clearance. The same holds for sAAPbeta, what happened to sAPPalpha for example. What about holo APP, etc..

Response to technical concern 2: We agree that using an internal control for normalization could be useful to consider non-specific changes in protein levels. However in all the studies assessing CSF Abeta levels that we are aware of (or any of the other key AD biomarkers) using human or mouse samples there has been no such normalization (Fagan Ann Neurol 2006, Bateman NEJM 2012, Mattson et al., Alzheimers Dement 2011). This is common practice in human studies and we believe that altering the methodology in mouse studies would potentially limit any translational implications of the findings. Even in the standardized protocol of the Alzheimer's Association for the quality control of CSF collection, processing, and measurement (e.g. Mattson et al., Alzheimers Dement 2011), which we try to follow as good as possible for the murine samples, there is no recommendation of normalizing the data to any reference protein. On the other hand the consortium suggests the use of a long-term quality control CSF sample for normalization purposes, something we have implemented in our measurements.

In contrast, brain Abeta and sAPPbeta levels have been normalized for the brain weight (results are reported as sAPPbeta levels per gram of wet brain) and again this matches what is commonly done in the field (e.g. Eketjäll et al J Neurosci 2013).

Technical concern 3: I am a little uncomfortable with the way the statistics is described, though the ANOVA with Dunnet's in Figure 2 is appropriate and perhaps the most important. However, in the methods it sounds as if a whole bunch of various methods were applied. I think it important to be clear on the a priori principal test and outcome versus what was post-hoc analysis.

Response to technical concern 3: We have now rephrased and clarified the statistical methods (manuscript page 15-16). In brief, the primary pre-specified analysis was whether there was a linear trend in CSF and brain Aβ levels depending on age. Additionally, to improve fit, a quadratic term was investigated exploratory. Only in case of significant linear trend subsequent special pairwise comparisons were done. This is in accordance with the principal of hierarchically ordered hypotheses. As pointed out by the reviewer, it was not intended a priori to test all pairwise comparisons between different time points and thus Dunnett's procedure was chosen to obtain maximum power and preserve the correct level of significance.

Technical concern 4: What happened in blood of mice? As these mice use the Thy-1 promoter blood might track better with CNS than other lines using promoters with peripheral expression. Potential translational impact would increase if the changes could be tracked in blood.

Response to technical concern 4: Indeed we have done the analysis of Abeta 40 and 42 in the plasma of APP23 mice (see Figure 2 below {unpublished data removed upon author request}). We observed that plasma Abeta 40 and 42 levels were steady until later stages, when plasma Abeta 42 exhibited a non-significant decline. The plasma Abeta 42/40 ratio declined significantly at 25 months when mice presented with a massive Abeta deposition in the brain. These data are in agreement with the observations in CSF but suggest a much lower sensitivity. From a biomarker perspective plasma Abeta levels and 42/40 ratio may hold potential to track disease progression but this may be blurred by peripherally generated Abeta in humans. Mechanistically, this observation may suggest that the brain to blood clearance is insufficient to compensate the pre-plaque Abeta generation (ceiling effect?). Unfortunately the blood of APP24 or APP51 mice was not available to run a similar analysis. For these reasons we have not included the APP23 data in the manuscript. If the editors feel appropriate we can add the APP23 blood data to the supplement.

2nd Editorial Decision 13 April 2015

Thank you for the submission of your revised manuscript to EMBO Molecular Medicine. We have now received the enclosed report and I am pleased to inform you that we will be able to accept your manuscript pending editorial final amendments.

Please submit your revised manuscript within two weeks. I look forward to seeing a revised form of your manuscript as soon as possible.

***** Reviewer's comments *****

Referee #1 (Comments on Novelty/Model System):

This remains a descriptive correlative study.

Referee #1 (Remarks):

The manuscript is changed little from the previous version. The clarifications on the statistical plan and evaluation are a good addition. Most of the rebuttal is simply that -a rebuttal- there is essentially no change in the data that is being included in the manuscript. The lack of a normalization protein in the CSF, the sparse data on mechanism of the increase in Abeta, and the limited impact that this will have on either interpretation of future human biomarker studies or planned biomarker studies makes me less enthusiastic than the other reviewers.
